# Liberal Versus Restrictive Fluid Therapy in the Early Management of Sepsis and Septic Shock: A Systematic Review

**DOI:** 10.7759/cureus.104913

**Published:** 2026-03-09

**Authors:** Cesar A Rojas-Landa, Jose R Flores-Valdes, Aldo Ascencio-Romo, Renée Guevara-Olivera, Sophia C Sánchez-Bertado, Karime Y Marquez-Prado, Mauricio Montelongo-Quevedo, Dulce M Mena-González, Diana C Reyes-Abundis, Benjamin A Rojas Landa

**Affiliations:** 1 General Practice, Universidad Autónoma de Baja California, Mexicali, MEX; 2 General Practice, Oncology Consultants PA, Houston, USA; 3 General Medicine, Universidad Autónoma del Estado de Hidalgo, Pachuca, MEX; 4 General Medicine, Instituto Tecnológico de Monterrey, Mexico City, MEX; 5 General Practice, Universidad Anáhuac México Sur, Mexico City, MEX; 6 General Medicine, Universidad Autónoma de México, Mexico City, MEX; 7 General Practice, Universidad Autónoma de Guadalajara, Guadalajara, MEX; 8 General Medicine, Universidad Autónoma de Guadalajara, Guadalajara, MEX; 9 General Medicine, Instituto Tecnológico de Monterrey, Monterrey, MEX; 10 Emergency Medicine, Instituto Mexicano del Seguro Social, Mexicali, MEX; 11 Emergency Medicine, Universidad Autónoma de Baja California, Mexicali, MEX

**Keywords:** fluid, fluid therapy, management, sepsis, septic shock

## Abstract

Sepsis and septic shock remain life-threatening conditions with high morbidity and mortality. Optimal fluid management during early resuscitation remains controversial, particularly regarding liberal versus restrictive strategies. This systematic review aimed to evaluate clinical outcomes associated with different intravenous (IV) fluid approaches in adult patients with sepsis and septic shock. A literature search was conducted in May 2024 using major medical databases. Observational cohort studies and randomized clinical trials assessing mortality and clinically relevant outcomes were included. Five studies met the inclusion criteria, comprising four retrospective cohort analyses and one multicenter randomized clinical trial. Observational data consistently demonstrated that higher cumulative fluid balance was independently associated with increased mortality and adverse outcomes, particularly after the initial resuscitation phase. However, randomized evidence did not demonstrate a significant difference in 90-day mortality between restrictive and liberal fluid strategies during early resuscitation. Overall, current evidence does not conclusively establish the superiority of one fixed fluid approach over another. These findings suggest that fluid management should be individualized and guided by patient physiology and dynamic clinical assessment rather than rigid volume thresholds. Further high-quality randomized studies are needed to clarify the optimal strategy.

## Introduction and background

Introduction

Background

Sepsis is a heterogeneous and life-threatening syndrome characterized by physiologic, pathologic, and biochemical abnormalities resulting from a dysregulated host response to infection [1-4]. The definition of sepsis has evolved significantly over the past decades, culminating in the Sepsis-3 consensus definition, which describes sepsis as life-threatening organ dysfunction, identified by an increase of ≥2 points in the Sequential Organ Failure Assessment (SOFA) score, secondary to infection [3,5-7]. Despite refinements in diagnostic criteria, sepsis remains associated with substantial morbidity, mortality, and healthcare burden worldwide. Reported in-hospital mortality ranges from 17% to 26%, increasing to approximately 45% in septic shock [8-10].

Early management following diagnosis is critical and traditionally focuses on three main principles: infection control, hemodynamic stabilization, and organ support [11]. Intravenous (IV) fluid administration remains a cornerstone of hemodynamic resuscitation. Current Surviving Sepsis Campaign (SSC) guidelines recommend administration of at least 30 mL/kg of intravenous crystalloid within the first three hours of recognition [12-15]. However, the certainty of evidence supporting this recommendation is low, and the optimal fluid volume during early resuscitation remains a topic of ongoing debate [16-18].

Rationale

Although significant advances have been made in understanding sepsis pathophysiology, uncertainty persists regarding the ideal fluid resuscitation strategy. The 2021 SSC guidelines downgraded the strength of the 30 mL/kg recommendation from strong to weak due to low-quality evidence [19,20], reflecting ongoing clinical equipoise. Some investigators have questioned whether a uniform fluid strategy is appropriate for all patients, particularly those at risk of fluid overload or with underlying cardiac or renal dysfunction [18].

Observational studies and randomized trials have suggested that a higher cumulative fluid balance may be associated with worse outcomes, including increased mortality and complications, whereas more restrictive or lower-volume approaches may not result in harm and may be associated with improved outcomes in certain subgroups [21-25]. Proposed mechanisms for harm from excessive fluid administration include delayed cardiovascular decompensation and organ dysfunction related to volume overload [26-29].

These considerations underscore the need to clarify whether liberal fluid resuscitation confers benefit or risk during the early phase of sepsis management. For the purpose of this review, early management refers to fluid administration during the initial 24 hours following sepsis or septic shock diagnosis.

In this review, “liberal fluid strategy” refers to protocols administering higher cumulative fluid volumes during early resuscitation, whereas “restrictive strategy” refers to protocols prioritizing earlier vasopressor use and lower cumulative fluid volumes after initial stabilization. Given variability in terminology across studies, we use the term “restrictive” to collectively describe lower-volume or conservative approaches.

The aim of this systematic review is to compare clinical outcomes, particularly short-term mortality, between liberal and restrictive fluid strategies in the early management of sepsis and septic shock.

## Review

Methods

Search Strategy

Bibliographic electronic databases, such as PubMed, Google Scholar, and Scopus, were used to search for articles in English and Spanish, filtering the year of publication between 2018 and 2024. The following keywords were used: “fluid therapy,” “restrictive fluid therapy,” “conservative fluid therapy,” “fluid therapy,” “sepsis,” “sepsis management,” and “septic shock” (Table 1).

**Table 1:** Search strategy

Search strategy	Results
("sepsis"[MeSH Terms] OR "sepsis"[Title/Abstract] OR "shock, septic"[MeSH Terms] OR "septic shock"[Title/Abstract]) AND ("liberal fluid therapy"[Title/Abstract] OR "conservative fluid therapy"[Title/Abstract] OR "fluid management"[Title/Abstract]) AND ("mortality"[MeSH Terms] OR "mortality"[Title/Abstract] OR "death"[Title/Abstract])	133
Search strategy	Results
sepsis AND septic shock AND liberal fluid therapy OR conservative fluid therapy AND mortality AND death AND randomized controlled trials	1,101

The protocol for this systematic review was prospectively registered in PROSPERO (CRD42024591836).

Inclusion and Exclusion Criteria

Types of study: We included randomized controlled trials (RCTs) and observational studies in humans in which different strategies of IV fluid interventions were implemented to manage sepsis. We excluded crossover trials, meta-analyses, and systematic reviews.

Only studies published in English were included.

Types of participants: Studies that included adult (>18 years of age) patients with sepsis or septic shock were considered. Sepsis was defined according to “The Third International Consensus Definitions for Sepsis and Septic Shock (Sepsis-3)”. Those that had patients with the following conditions were excluded: pregnancy, life-threatening bleeding, acute burn injury, trauma, cardiogenic pulmonary edema, stroke, or epilepsy.

Types of intervention: We searched for articles whose intervention focused on liberal fluid management in patients with sepsis. In addition, we also considered articles that focused on or compared the intervention with restrictive fluid management, to achieve a comparison with these two types of interventions, the management, complications, benefits, and, above all, the impact on the mortality rate of patients with sepsis and septic shock.

Early management was defined as fluid administration occurring within the first 24 hours following diagnosis of sepsis or septic shock, consistent with early resuscitation protocols described in international guidelines.

Outcomes: In this systematic review, we aim to evaluate the impact of liberal versus restrictive fluid therapy in the early management of sepsis and septic shock. The primary outcome was mortality, which reflected the ultimate effectiveness of the treatment strategy.

The primary outcome was short-term mortality (28-day, 30-day, or 90-day mortality depending on individual study reporting). When multiple mortality endpoints were available, the earliest clinically relevant timepoint was extracted.

Selection of studies: Following an initial screening based on the title and abstract, two reviewers (SCSB and RGO) independently selected trials for inclusion in this review using predetermined inclusion and exclusion criteria. We resolved disagreements about including studies by consensus and consultation with a third review author (MMQ). For full-text screening, two reviewers (DMMG and AAR) independently selected trials for inclusion in this review using predetermined inclusion and exclusion criteria. We resolved disagreements on study inclusion by consensus and consultation with a third review author (MMQ).

Statistical synthesis: A quantitative meta-analysis was not performed due to significant clinical and methodological heterogeneity across studies, including variability in definitions of liberal and restrictive strategies, differences in mortality endpoints, study designs (observational versus randomized), and patient populations. Therefore, a structured narrative synthesis was conducted.

Risk of Bias Assessment

Eligible RCTs and observational studies underwent quality control and risk of bias assessment using the Newcastle-Ottawa Scale for those that were cohorts and case-control studies, and Cochrane risk-of-bias version 2 (RoB2.0) for those that were randomized controlled trials. In addition, robvis, a web app, was required to visualize the results as bias graphs [30-32].

Quality control and bias assessment were conducted independently by two reviewers (RGO and GRT), and disagreements were resolved in consensus and consultation with a third review author (DMMG).

Results

Literature Search

We conducted a systematic literature review to assess and compare outcomes related to fluid therapy strategies during the early resuscitation of patients with sepsis and septic shock, focusing on restrictive and liberal approaches and their association with outcomes such as mortality and Sequential Organ Failure Assessment (SOFA) score. The search strategy was performed in May 2024 using three electronic databases: PubMed, Google Scholar, and Scopus. A combination of keywords and search terms was used, including “sepsis,” “septic shock,” “fluid management,” “liberal fluid therapy,” “restrictive fluid therapy,” “mortality,” and “death.”

**Figure 1:**
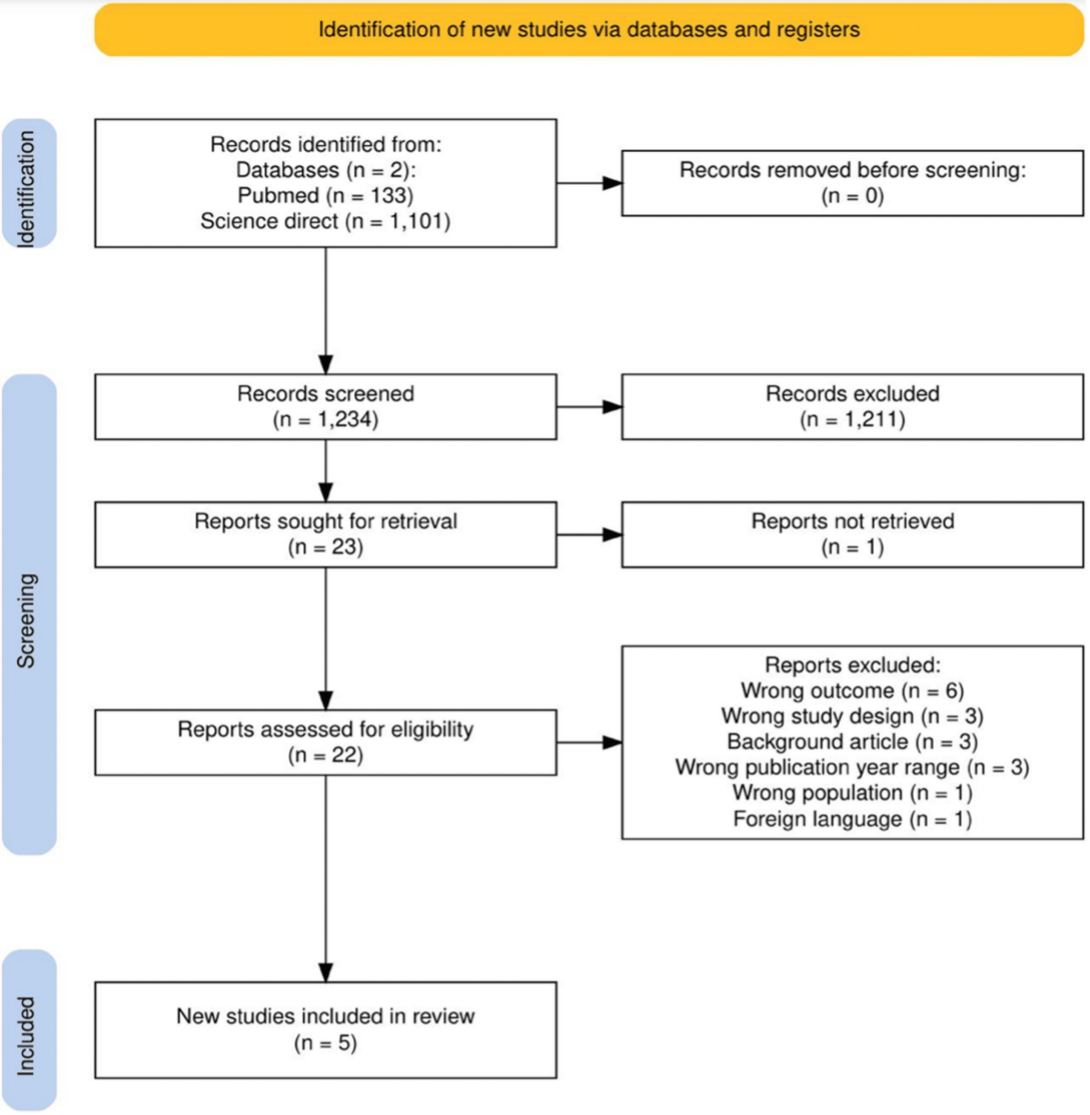
Flowchart showing search results and screening of all included studies At the end of screening, five studies were included.

A total of 1,234 records were identified through database searching. After title and abstract screening, 1,211 records were excluded. Twenty-three reports were assessed for full-text eligibility, of which one report could not be retrieved. Of the remaining 22 reports, 17 were excluded after full-text review. Finally, five studies met the inclusion criteria and were included in this systematic review. Figure 1 illustrates the study selection process following the Preferred Reporting Items for Systematic Reviews and Meta-Analyses (PRISMA) flow diagram.

To achieve a critical assessment of the quality of the selected studies (cases, controls, and cohorts), it was necessary to subject them to a subjective evaluation using a standardized questionnaire, the Newcastle-Ottawa Quality Assessment Scale. Through the cumulative scoring across the three domains of this scale, the quality of each study was categorized as good, fair, or poor. The results of this assessment are presented in Table 2.

**Table 2:** Newcastle-Ottawa Scale for case-control and cohort studies’ risk of bias appraisal

Author, year	Study design	Selection	Comparability	Outcome/exposure	Total	Subjective evaluation
Zhang et al. [33]	Cohort study	4	1	3	8	Good quality
van Mourik et al. [34]	Cohort study	4	1	2	7	Good quality
Wang et al. [35]	Retrospective cohort study	3	1	3	7	Good quality
Wang and Feng [36]	Retrospective observational cohort study	4	2	3	9	Good quality

**Figure 2:**
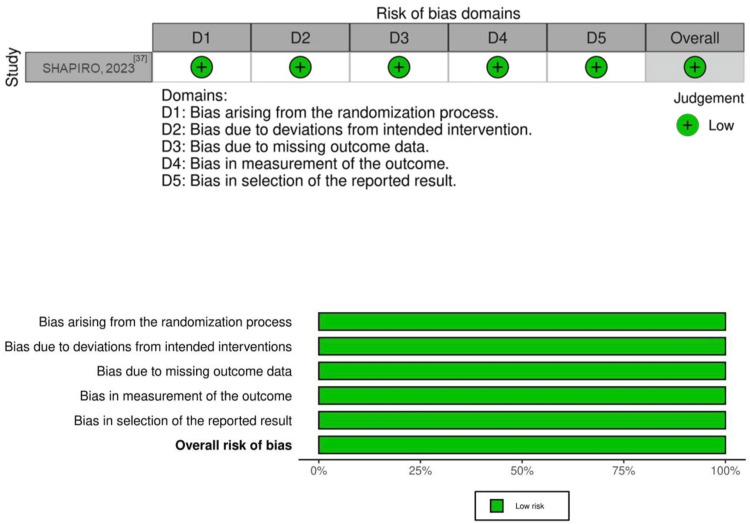
“Traffic light” plots of domain-level judgments for each individual outcome using the web app tool, robvis

To achieve a critical assessment of the quality of the selected randomized controlled trials, it was necessary to subject them to a subjective evaluation using a standardized questionnaire from Cochrane’s risk-of-bias version 2 (RoB 2.0). Through qualitative assessment across the five domains comprising this tool, the overall risk of bias for each study was determined, categorized as low, some concerns, or high. The results of this assessment are presented in Figure 2.

Study Characteristics

There was variation in study design, patient selection methods, population size, and reported endpoints across the included articles. Three studies were conducted in China, one in the Netherlands, and one in the United States. Population sizes ranged from 322 to 22,868 patients. Three studies were published in 2020, one in 2022, and one in 2023.

Regarding study design, four studies were retrospective observational cohort analyses, including one that applied a dynamic treatment regimen model to a large intensive care unit (ICU) database, and one was a multicenter randomized clinical trial. Most studies (n = 3) defined sepsis according to the Sepsis-3 criteria (Third International Consensus Definitions for Sepsis and Septic Shock), one used the Sepsis-2 definition, and one did not clearly specify the diagnostic criteria.

Among the studies conducted in China, the reported mean age was 67.40 ± 16.57 years in the 2022 study and approximately 62 years in the 2020 cohort. In the US-based randomized clinical trial, the mean age was 59.5 ± 15.9 years. The most frequently reported comorbidities across studies included hypertension, diabetes mellitus, chronic kidney disease, and heart failure.

Clinical Outcomes

Mortality rates varied across the included studies due to differences in study design and population characteristics. In the large retrospective cohort by Zhang et al., 3,820 deaths were reported among 22,868 patients (16.5%) [33]. van Mourik et al. reported an ICU mortality rate of 20% among patients with septic shock after shock reversal [34]. In the acute respiratory distress syndrome (ARDS) cohort by Wang et al., in-hospital mortality among patients with sepsis complicated by ARDS reached 69% [35]. In the cohort study by Wang and Feng, the reported 30-day mortality rate was 40.76% [36]. However, this study evaluated the prognostic value of fluid balance variables in predicting mortality among patients with sepsis rather than directly comparing liberal and restrictive fluid resuscitation strategies. Therefore, its findings contribute evidence regarding the association between fluid balance and outcomes but do not represent a direct comparison of fluid therapy strategies.

The Crystalloid Liberal or Vasopressors Early Resuscitation in Sepsis (CLOVERS) randomized clinical trial by Shapiro et al. reported 90-day mortality rates of 14% in the restrictive group and 14.9% in the liberal group [37].

Observational data consistently demonstrated an association between higher cumulative fluid balance and worse clinical outcomes. van Mourik et al. reported that for every 10 mL/kg increase in daily fluid balance after shock reversal, the odds of ICU mortality increased (odds ratio (OR): 3.18, 95% confidence interval (CI): 1.90-5.32) [34]. Similarly, Wang et al. found that a higher mean daily fluid balance was independently associated with increased mortality in septic patients complicated by ARDS, whereas survivors exhibited lower fluid input and higher output, suggesting a potential benefit of negative fluid balance during later phases of resuscitation [35].

Zhang et al., using a dynamic treatment regimen model, demonstrated that an individualized approach favoring lower fluid volumes when clinically appropriate was associated with longer modeled survival time (5.7 versus 4.1 days; p < 0.001) [33]. However, this was derived from predictive modeling rather than a direct interventional comparison.

In contrast, the multicenter CLOVERS randomized trial found no statistically significant difference in 90-day mortality between restrictive and liberal fluid strategies, despite clear differences in fluid administration and vasopressor use [37].

Overall, while observational evidence suggests that positive fluid balance is associated with increased mortality and adverse outcomes, the largest randomized trial to date did not demonstrate a mortality benefit of a restrictive strategy over a liberal approach during early resuscitation.

Table 3 summarizes the study-specific fluid strategy definitions, mortality outcomes, and key findings from each included study.

**Table 3:** General outcomes ARDS: acute respiratory distress syndrome, ICU: intensive care unit, OR: odds ratio, CI: confidence interval, AUC: area under the curve, IV: intravenous

Study	Study design	Population (number)	Fluid strategy (study-specific definition)	Primary outcome	Main mortality findings	Other relevant outcomes
Zhang et al. [33]	Retrospective cohort with dynamic treatment regimen model	Sepsis (22,868 ICU patients)	Liberal ≥40 mL/kg/day versus restrictive <40 mL/kg/day (evaluated on day 1, 3, and 5)	Survival time	Optimal (more restrictive) strategy associated with longer survival (5.7 versus 4.1 days; p < 0.001)	Proportion of inappropriately liberal-treated patients increased from 19.3% (day 1) to 29.5% (day 5)
van Mourik et al. [34]	Retrospective observational cohort	Septic shock after shock reversal (636 patients)	Daily and cumulative fluid balance after shock reversal (per 10 mL/kg increase)	ICU mortality	Higher daily fluid balance independently associated with ICU mortality (OR: 3.18 (1.90-5.32) per 10 mL/kg increase)	Increased 30-day, 90-day, hospital, and one-year mortality with higher fluid balance
Wang et al. [35]	Retrospective observational cohort	Sepsis with ARDS (84 patients)	Mean daily fluid balance over seven days	In-hospital mortality	Higher mean daily fluid balance independently associated with mortality	Survivors had lower fluid input and higher output; early negative balance associated with better prognosis
Wang and Feng [36]	Retrospective observational cohort study evaluating prognostic value of fluid balance	Sepsis (1,185 ICU patients)	Total fluid input and output in first 24 hours (model-based analysis)	30-day mortality	Inclusion of IV fluid variables improved mortality prediction (AUC: 0.781 testing set)	Demonstrated prognostic value of fluid balance variables
Shapiro et al. (CLOVERS trial) [37]	Multicenter randomized controlled trial	Sepsis-induced hypotension (1,563 patients)	Restrictive strategy (early vasopressors, lower IV fluids) versus liberal strategy (higher IV fluids before vasopressors), 24-hour protocol	90-day mortality before discharge home	No significant difference: 14.0% (restrictive) versus 14.9% (liberal); difference: -0.9% (95% CI: -4.4 to 2.6); p = 0.61	Similar rates of serious adverse events between groups

Discussion

This systematic review aimed to identify the optimal intravenous (IV) fluid therapy strategy for hemodynamic stabilization in patients with sepsis and septic shock, comparing liberal and restrictive fluid resuscitation approaches. The findings of the included studies provide a comprehensive overview of current evidence regarding fluid management in this critically ill population.

The available evidence suggests a potential association between higher cumulative fluid balance and increased mortality, as well as a greater incidence of adverse events related to fluid overload. Several studies reported that a more restrictive fluid strategy during the post-resuscitation phase may be associated with improved survival and reduced complications such as pulmonary edema, cerebral edema, coagulopathy, and impaired renal recovery after hospital discharge [38,39]. In particular, patients with a higher fluid balance were found to have a 3.18-fold increased mortality risk compared to those with a lower balance [34]. Zhang et al. observed that the proportion of patients receiving liberal fluid management (>40 mL/kg/d) who might have experienced longer survival under a restrictive approach (<40 mL/kg/d) increased from 19.3% on day 1 to 29.5% by day 5. Moreover, optimizing fluid strategy was associated with a statistically significant extension in survival time (5.7 (2.0, 5.9) days versus 4.1 (2.0, 5.0) days; p < 0.001) [33]. These findings underscore the dynamic nature of fluid requirements and suggest that treatment strategies should be individualized according to patient response, including parameters such as urine output and evolving hemodynamic status.

However, not all studies demonstrated a consistent association between initial fluid volume and mortality. Wang et al. [35] and Shapiro et al. [37], both including larger patient cohorts, reported no significant relationship between early fluid administration and 90-day mortality. These discrepancies may be attributable to differences in study design, timing of fluid balance assessment, patient severity, and definitions of liberal versus restrictive strategies. Furthermore, the predominance of observational designs among the included studies limits the ability to infer causality and raises the possibility of residual confounding, particularly confounding by indication.

Interestingly, some evidence suggests that an initial adequate fluid resuscitation followed by a restrictive or deresuscitative strategy in later phases may provide clinical benefit in selected patients with severe sepsis or septic shock. This concept aligns with the evolving understanding that fluid therapy should be viewed as a dynamic process rather than a fixed-volume intervention. Nevertheless, this observation was primarily derived from a single study involving patients with sepsis complicated by acute respiratory distress syndrome (ARDS) [36], limiting its generalizability. Further randomized controlled trials are needed to clarify the optimal timing and volume thresholds for transitioning from resuscitation to restriction.

The strengths of this review include a structured and comprehensive synthesis of available evidence comparing liberal and restrictive fluid strategies, as well as the correlation of fluid balance with clinically relevant outcomes such as mortality and major adverse events. By systematically evaluating the current literature, this review contributes to a more nuanced understanding of fluid management in sepsis and septic shock.

Despite the inclusion of a substantial number of patients, several limitations must be acknowledged. The relatively small number of eligible studies and the restriction to patients from a limited number of countries reduce the external validity of the findings. Additionally, heterogeneity in study design, patient populations, fluid definitions, and outcome measures precluded definitive conclusions. The predominance of observational data further limits causal inference. Therefore, while the evidence suggests that excessive positive fluid balance may be associated with worse outcomes, there is insufficient high-quality evidence to definitively endorse a universally restrictive or liberal strategy. Future research should prioritize large, well-designed randomized controlled trials and aim to evaluate long-term outcomes to better define individualized fluid management strategies in sepsis and septic shock.

## Conclusions

Sepsis and septic shock remain life-threatening conditions with high morbidity and mortality. Across the included studies, higher cumulative fluid balance was consistently associated with worse clinical outcomes in observational cohorts, particularly after the initial resuscitation phase. However, the largest randomized clinical trial to date did not demonstrate a significant mortality difference between restrictive and liberal fluid strategies during early resuscitation.

Taken together, current evidence does not conclusively establish the superiority of one fixed fluid strategy over another. These findings highlight the complexity of fluid resuscitation in sepsis and suggest that fluid management should be individualized, guided by dynamic clinical assessment and patient physiology rather than rigid volume thresholds. Further high-quality randomized trials are needed to clarify the optimal approach to fluid therapy in this population.

## References

[1] Liu D, Huang SY, Sun JH (2022). Sepsis-induced immunosuppression: mechanisms, diagnosis and current treatment options. Mil Med Res.

[2] Purcarea A, Sovaila S (2020). Sepsis, a 2020 review for the internist. Rom J Intern Med.

[3] Singer M, Deutschman CS, Seymour CW (2016). The Third International Consensus Definitions for Sepsis and Septic Shock (Sepsis-3). JAMA.

[4] Bone RC, Balk RA, Cerra FB (1992). Definitions for sepsis and organ failure and guidelines for the use of innovative therapies in sepsis. The ACCP/SCCM Consensus Conference Committee. American College of Chest Physicians/Society of Critical Care Medicine. Chest.

[5] Shankar-Hari M, Phillips GS, Levy ML (2016). Developing a new definition and assessing new clinical criteria for septic shock: for the Third International Consensus Definitions for Sepsis and Septic Shock (Sepsis-3). JAMA.

[6] Seymour CW, Liu VX, Iwashyna TJ (2016). Assessment of clinical criteria for sepsis: for the Third International Consensus Definitions for Sepsis and Septic Shock (Sepsis-3). JAMA.

[7] Font MD, Thyagarajan B, Khanna AK (2020). Sepsis and septic shock - basics of diagnosis, pathophysiology and clinical decision making. Med Clin North Am.

[8] Arefian H, Heublein S, Scherag A (2017). Hospital-related cost of sepsis: a systematic review. J Infect.

[9] Fleischmann C, Scherag A, Adhikari NK (2016). Assessment of global incidence and mortality of hospital-treated sepsis. Current estimates and limitations. Am J Respir Crit Care Med.

[10] Holst LB, Haase N, Wetterslev J (2014). Lower versus higher hemoglobin threshold for transfusion in septic shock. N Engl J Med.

[11] Hotchkiss RS, Moldawer LL, Opal SM, Reinhart K, Turnbull IR, Vincent JL (2016). Sepsis and septic shock. Nat Rev Dis Primers.

[12] Srzić I, Nesek Adam V, Tunjić Pejak D (2022). Sepsis definition: what’s new in the treatment guidelines. Acta Clin Croat.

[13] Guarino M, Perna B, Cesaro AE, Maritati M, Spampinato MD, Contini C, De Giorgio R (2023). 2023 update on sepsis and septic shock in adult patients: management in the emergency department. J Clin Med.

[14] Oczkowski S, Alshamsi F, Belley-Cote E, Centofanti JE, Hylander Møller M, Nunnaly ME, Alhazzani W (2022). Surviving Sepsis Campaign Guidelines 2021: highlights for the practicing clinician. Pol Arch Intern Med.

[15] Prescott HC, Ostermann M (2023). What is new and different in the 2021 Surviving Sepsis Campaign guidelines. Med Klin Intensivmed Notfmed.

[16] Seitz KP, Qian ET, Semler MW (2022). Intravenous fluid therapy in sepsis. Nutr Clin Pract.

[17] Gavelli F, Castello LM, Avanzi GC (2021). Management of sepsis and septic shock in the emergency department. Intern Emerg Med.

[18] Jozwiak M, Hamzaoui O, Monnet X, Teboul JL (2018). Fluid resuscitation during early sepsis: a need for individualization. Minerva Anestesiol.

[19] Evans L, Rhodes A, Alhazzani W (2021). Surviving Sepsis Campaign: international guidelines for management of sepsis and septic shock 2021. Intensive Care Med.

[20] Rhodes A, Evans LE, Alhazzani W (2017). Surviving Sepsis Campaign: international guidelines for management of sepsis and septic shock: 2016. Intensive Care Med.

[21] Acheampong A, Vincent JL (2015). A positive fluid balance is an independent prognostic factor in patients with sepsis. Crit Care.

[22] Corl KA, Prodromou M, Merchant RC (2019). The Restrictive IV Fluid Trial in Severe Sepsis and Septic Shock (RIFTS): a randomized pilot study. Crit Care Med.

[23] Brown RM, Semler MW (2019). Fluid management in sepsis. J Intensive Care Med.

[24] Wang HL, Shao J, Liu WL, Wu F, Chen HB, Zheng RQ, Chen QH (2021). Initial fluid resuscitation (30 mL/kg) in patients with septic shock: more or less?. Am J Emerg Med.

[25] Macdonald S (2022). Fluid resuscitation in patients presenting with sepsis: current insights. Open Access Emerg Med.

[26] Marik PE, Byrne L, van Haren F (2020). Fluid resuscitation in sepsis: the great 30 mL per kg hoax. J Thorac Dis.

[27] Sadaka F, Juarez M, Naydenov S, O'Brien J (2014). Fluid resuscitation in septic shock: the effect of increasing fluid balance on mortality. J Intensive Care Med.

[28] Samoni S, Vigo V, Reséndiz LI (2016). Impact of hyperhydration on the mortality risk in critically ill patients admitted in intensive care units: comparison between bioelectrical impedance vector analysis and cumulative fluid balance recording. Crit Care.

[29] Maitland K, George EC, Evans JA (2013). Exploring mechanisms of excess mortality with early fluid resuscitation: insights from the FEAST trial. BMC Med.

[30] Sterne JA, Savović J, Page MJ (2019). RoB 2: a revised tool for assessing risk of bias in randomised trials. BMJ.

[31] Wells GA, Shea BJ, O' Connell D, Peterson J, Welch V, Losos M, Tugwell P (2013). The Newcastle-Ottawa Scale (Nos) for Assessing the Quality of Non-randomized Studies in Meta-Analysis. Internet.

[32] McGuinness LA, Higgins JP (2021). Risk-of-bias VISualization (robvis): an R package and Shiny web app for visualizing risk-of-bias assessments. Res Synth Methods.

[33] Zhang Z, Zheng B, Liu N (2020). Individualized fluid administration for critically ill patients with sepsis with an interpretable dynamic treatment regimen model. Sci Rep.

[34] van Mourik N, Geerts BF, Binnekade JM (2020). A higher fluid balance in the days after septic shock reversal is associated with increased mortality: an observational cohort study. Crit Care Explor.

[35] Wang YM, Zheng YJ, Chen Y (2020). Effects of fluid balance on prognosis of acute respiratory distress syndrome patients secondary to sepsis. World J Emerg Med.

[36] Wang Y, Feng S (2022). A prediction model for 30-day mortality of sepsis patients based on intravenous fluids and electrolytes. Medicine (Baltimore).

[37] Shapiro NI, Douglas IS, Brower RG (2023). Early restrictive or liberal fluid management for sepsis-induced hypotension. N Engl J Med.

[38] Woodward CW, Lambert J, Ortiz-Soriano V (2019). Fluid overload associates with major adverse kidney events in critically ill patients with acute kidney injury requiring continuous renal replacement therapy. Crit Care Med.

[39] Self WH, Semler MW, Bellomo R (2018). Liberal versus restrictive intravenous fluid therapy for early septic shock: rationale for a randomized trial. Ann Emerg Med.

